# Improving weight loss among young adults with excess body weight using a theory-driven digital self-regulation program: a randomized controlled trial

**DOI:** 10.1093/abm/kaag017

**Published:** 2026-04-19

**Authors:** Han Shi Jocelyn Chew, Jia Wen Ngooi, Wen Wei Ang, Ruo Chen Du, Pin Zhong Chan, Miia Jansson, Bin Zhu, Yu Cao, Chong Wah Ngo, Roger Foo, Asim Shabbir, Dean Ho, Nick Sevdalis, Kee Yuan Ngiam

**Affiliations:** Alice Lee Centre for Nursing Studies, Yong Loo Lin School of Medicine, National University of Singapore, Singapore 117599, Singapore; Cardiovascular Metabolic Disease Translational Research Programme, Yong Loo Lin School of Medicine, National University of Singapore, Singapore 117599, Singapore; Department of Biomedical Informatics, Yong Loo Lin School of Medicine, National University of Singapore, Singapore 117599, Singapore; Centre for Behavioral and Implementation Science Interventions (BISI), Yong Loo Lin School of Medicine, National University of Singapore, Singapore 117599, Singapore; Alice Lee Centre for Nursing Studies, Yong Loo Lin School of Medicine, National University of Singapore, Singapore 117599, Singapore; Alice Lee Centre for Nursing Studies, Yong Loo Lin School of Medicine, National University of Singapore, Singapore 117599, Singapore; Department of Biostatistics, Yong Loo Lin School of Medicine, National University of Singapore, Singapore 117599, Singapore; Department of Nursing, Ng Teng Fong General Hospital, 1 Jurong East Street 21, Singapore 609606, Singapore; Research Unit of Health Sciences and Technology, Faculty of Medicine, University of Oulu, Oulu, Oulu 90220, Finland; School of Computing and Information Systems, Singapore Management University, Singapore 178902, Singapore; School of Computing and Information Systems, Singapore Management University, Singapore 178902, Singapore; School of Computing and Information Systems, Singapore Management University, Singapore 178902, Singapore; Cardiovascular Metabolic Disease Translational Research Programme, Yong Loo Lin School of Medicine, National University of Singapore, Singapore 117599, Singapore; Department of Cardiology, National University Heart Centre, Singapore 119074, Singapore; Department of Surgery, National University Hospital, Singapore 119074, Singapore; Department of Biomedical Engineering, National University of Singapore, Singapore 117599, Singapore; Centre for Behavioral and Implementation Science Interventions (BISI), Yong Loo Lin School of Medicine, National University of Singapore, Singapore 117599, Singapore; Department of Biomedical Informatics, Yong Loo Lin School of Medicine, National University of Singapore, Singapore 117599, Singapore; Department of Surgery, National University Hospital, Singapore 119074, Singapore

**Keywords:** weight loss, overweight, obesity, artificial intelligence, mobile applications, randomized controlled trial

## Abstract

**Background:**

Lifestyle modification is fundamental for obesity management but remains challenging.

**Purpose:**

To test the effectiveness of a 12-week digital self-regulation program on improving weight loss and self-regulation among young adults.

**Methods:**

A 2-arm parallel 1:1 randomized controlled trial was conducted. Participants were randomly assigned to either the intervention or the control group. The primary outcomes were weight and self-regulation, while the secondary outcomes were nutrition knowledge, eating phenotypes, overeating habits, anxiety, and depression. All data were collected at weeks 0, 12, and 24.

**Results:**

We showed the effectiveness of a digital commitment device on body weight (−1.90 ± 6.4 kg, *P *= .002), body mass index (−0.45 ± 2.0 kg/m^2^, *P *= .002), waist circumference (−2.10 ± 12.1 cm, *P *= .01), basal metabolic rate (−20.30 ± 118.6 kcal, *P *= .04) and body roundness index (−0.17 ± 0.6, *P *= .014). Interventional effects were sustained for waist circumference (−0.8 ± 2.8 cm, *P *= .05) and body roundness index (−0.07 ± 0.36, *P *= .047) 3 months beyond removing access to the commitment device. General improvements in nutrition knowledge, psychological flexibility, uncontrolled eating, and anxiety were found among both groups, but differences were only significant for depression scores (−0.06 ± 1.5, *P *= .032) and eating self-regulation (0.16 ± 0.54, *P *= .003).

**Conclusion:**

This study showed the potential of a digital commitment device in improving eating self-regulation, anthropometry, and mental wellness for scalable implementation in the community setting.

**Trial Registration:**

Clinicaltrials.gov (NCT05969639).

## Introduction

By 2035, more than half of the global population is expected to be overweight/obese, costing approximately $4.32 trillion in disease burden.^1^ This challenge is further compounded by the rising prevalence of overweight and obesity among young adults, reflecting a shift toward earlier onset of excess weight and prolonged exposure to cardiometabolic risk across the life course (World Health Organization, 2022). While clinical obesity management support exists, studies suggest that individuals with excess body weight often prefer self-management approaches for weight loss.^2^^,^^3^ However, weight loss often fails due to the lack of self-regulation, which refers to the cognitive control over thoughts, feelings, and behaviors—a resource that can be depleted with time.^4^ In obesogenic environments characterized by persistent food cues, stress, and competing demands, self-regulatory resources are repeatedly taxed and may become depleted over time.^5^

Prominent self-regulation theories, including the self-control strength model,^6^ social cognitive theory of self-regulation,^7^ dual-process models,^8^ cybernetic model,^9^ and temporal self-regulation theory (TST),^10^ converge on the premise that successful lifestyle behavior change depends on a core set of self-regulatory skills. These rudiments include goal setting, self-monitoring, and self-reflection.^11^^,^^12^ However, self-regulation skills and capacity that are crucial for impulse control (eg, controlling food temptations) such as inhibitory control, delayed gratification, and the ability to align immediate decisions with distal health outcomes are less well-specified and more difficult to address. Correspondingly, many existing lifestyle interventions prioritize education and self-management, placing limited emphasis on strengthening self-regulatory skills and capacity for impulse control, which may partly explain their modest and short-lived effects.

In our current digitalization era, high smartphone penetration presents an opportunity to provide convenient behavior change interventions through smartphone apps. For example, self-regulation mobile applications such as the eCHANGE app,^13^ the Oviva Direkt app,^14^ and the Lose it app^15^ have been shown to support weight management by improving self-monitoring. However, a few such as the Balance It app^16^ focus on improving self-regulation through activities that promote self-reflection, formulation of if-then plans, provision of goal-linked prompts, and gamification to reinforce certain positive behaviors.^17^^,^^18^ Moreover, few studies have focused on the young adult population, a group often neglected in preventive health interventions, but at a critical period for the development of lifelong healthy eating habits.^19^^,^^20^ Even fewer studied the mechanism behind the effectiveness of digital health interventions for weight loss.

Considering the gaps in various self-regulation theories, the TST is a relatively new theory that focuses on the intention-behavior gap, which is posited to be influenced by time perspective (ie, extend of consideration of future consequences), behavioral prepotency (ie, habits), and self-regulation capacity (ie, ability to self-regulate thoughts, feelings, and behaviors).^10^ This requires interventions to go beyond standard self-monitoring functions such as calorie or weight logging to focus on developing self-regulation skills such as inhibitory control, delayed gratification, and the ability to align immediate decisions with distal health outcomes. These theoretical components are hypothesized to be interrelated mechanisms through which the intervention may influence weight change, which could be investigated through association analyses.

Through this study, we aimed to test the effectiveness of a 12-week digital self-regulation program on improving weight loss and self-regulation among young adults. We hypothesized that participants in the intervention group would demonstrate greater improvements in weight loss and self-regulation as compared to participants in the control group at baseline, week 12, and week 24. Our secondary aim is to uncover the mechanism behind eating habits, with a hypothesis that there is an association between weight, self-regulation, eating phenotype, time perspective, habit of overeating, app engagement, anxiety, and depression.

## Methods

### Study design and sampling method

**Figure 1:**
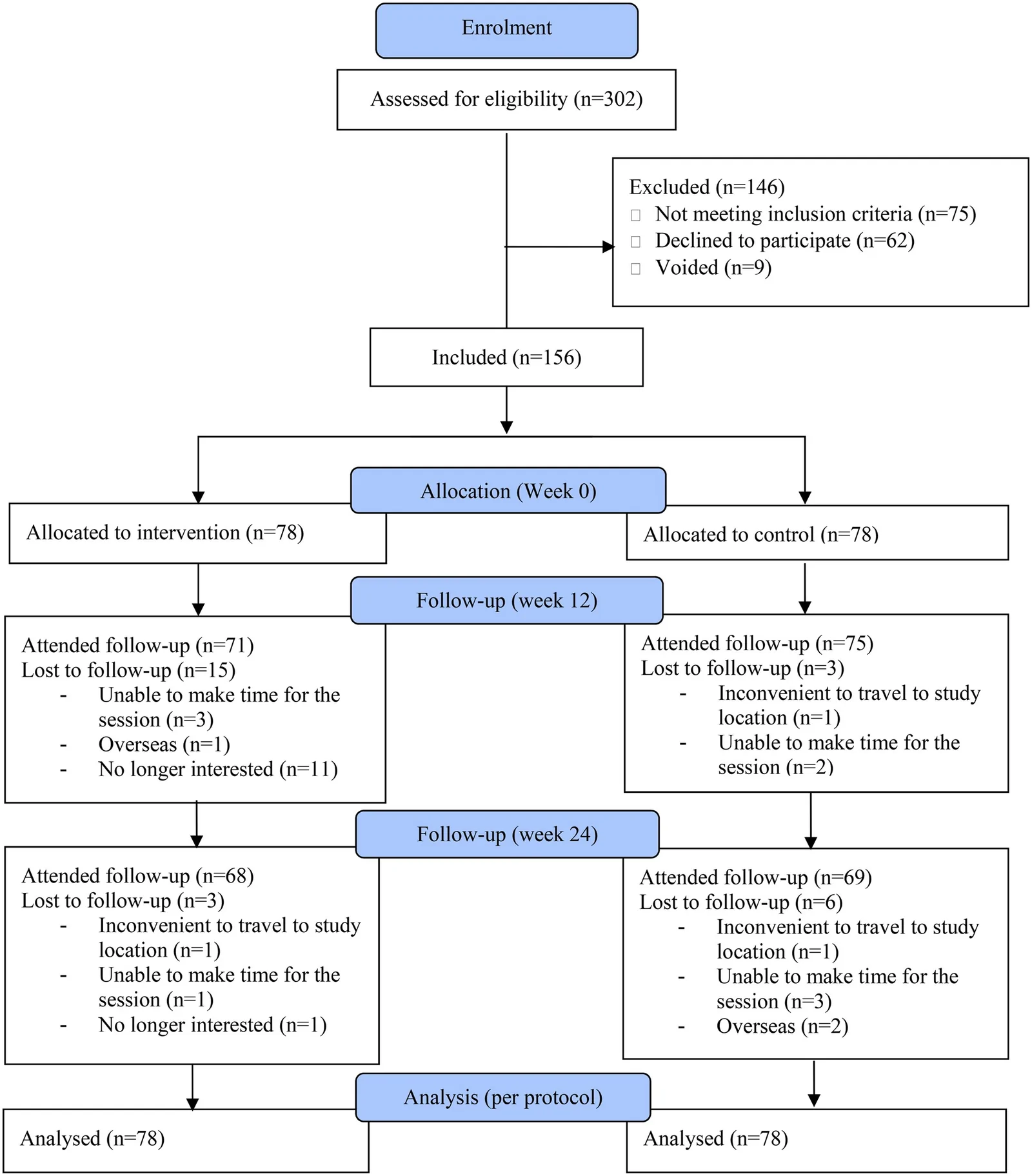
Consort flow diagram.

A 2-arm parallel 1:1 randomized controlled trial was conducted and reported according to the CONSORT-Outcomes 2022 Extension^21^ reporting guideline alongside the template for intervention description and replication (TIDieR) checklist (Figure 1).^22^ Participants were recruited using convenience sampling from the community through social media advertisements (eg, telegram). Potential participants were included if they (1) had a body mass index (BMI) ≥23 kg/m^2^ (World Health Organization’s recommended cut-off for the Asian population)^23^; (2) were between the ages of 21 and 35 years old; (3) could understand and read the English language; (4) used a smartphone that could download apps; and (5) were willing to travel to the National University of Singapore for body composition analysis. Participants were excluded if they (1) were pregnant or lactating, (2) were participating in a structured weight loss program, (3) were planning or receiving pharmacotherapy or bariatric surgery within the next 6 months, and (4) were diagnosed with a severe mental disorder.

### Sample size

Based on a power analysis using the G* power 3.1 software,^24^ at least 150 participants were needed for the study to have an 80% power and 2-tailed significance level of .05 to detect a group difference of 2.5 kg (SD 5.4) at week 12.^25^

### Procedure

Participants were recruited via social media channels (ie, Telegram) and posters placed around a university, where potential participants registered their interest in participating in the study by contacting the contact details on the social media advertisement. The research assistant then reached out to them to explain the study procedures in detail and collected written informed consent through an online video meeting. Next, participants underwent in-person anthropometric measurements at the National University of Singapore and completed a set of online questionnaires through the university-endorsed survey platform (Qualtrics) on their sociodemographic profile, self-regulation of eating behavior, overeating habit, eating phenotype, nutrition knowledge, and psychological flexibility. Data collection of various anthropometric measurements was conducted by a research assistant (J.W.N.) trained by the principal investigator (PI, H.S.J.C.) through repeated demonstrations. Participants were then randomly assigned to either the intervention or control group using a computer-generated random list of numbers generated by the PI, who was not involved in the data collection process to maintain allocation concealment. Participants in the intervention group were onboarded to a novel 12-week behavior change program called the Eating Trigger Response Inhibition program 2.0 (eTRIP 2.0), where the research assistant assisted participants in downloading the app and creating an account to access and use the app. Access to the app was ceased at week 12 for participants in the intervention group. Both groups were required to return for follow-up sessions at weeks 12 and 24, where their anthropometric measurement data were collected by a research assistant, similar to how it was done in the first session. Participants were reimbursed $10 for completing each follow-up session.

### Ethical consideration

This study was approved by the National University of Singapore Institutional Review Board (NUS-IRB-2022-650). This study conformed to the ethical guidelines of the 1975 Declaration of Helsinki. Written informed consent was obtained from each participant; the study data were de-identified with subject codes, and participants were reimbursed for their time.

### Control group

Participants in the control group did not receive any active intervention besides body composition reports at weeks 0, 12, and 24.

### Intervention group: eTRIP 2.0

**Figure 2:**
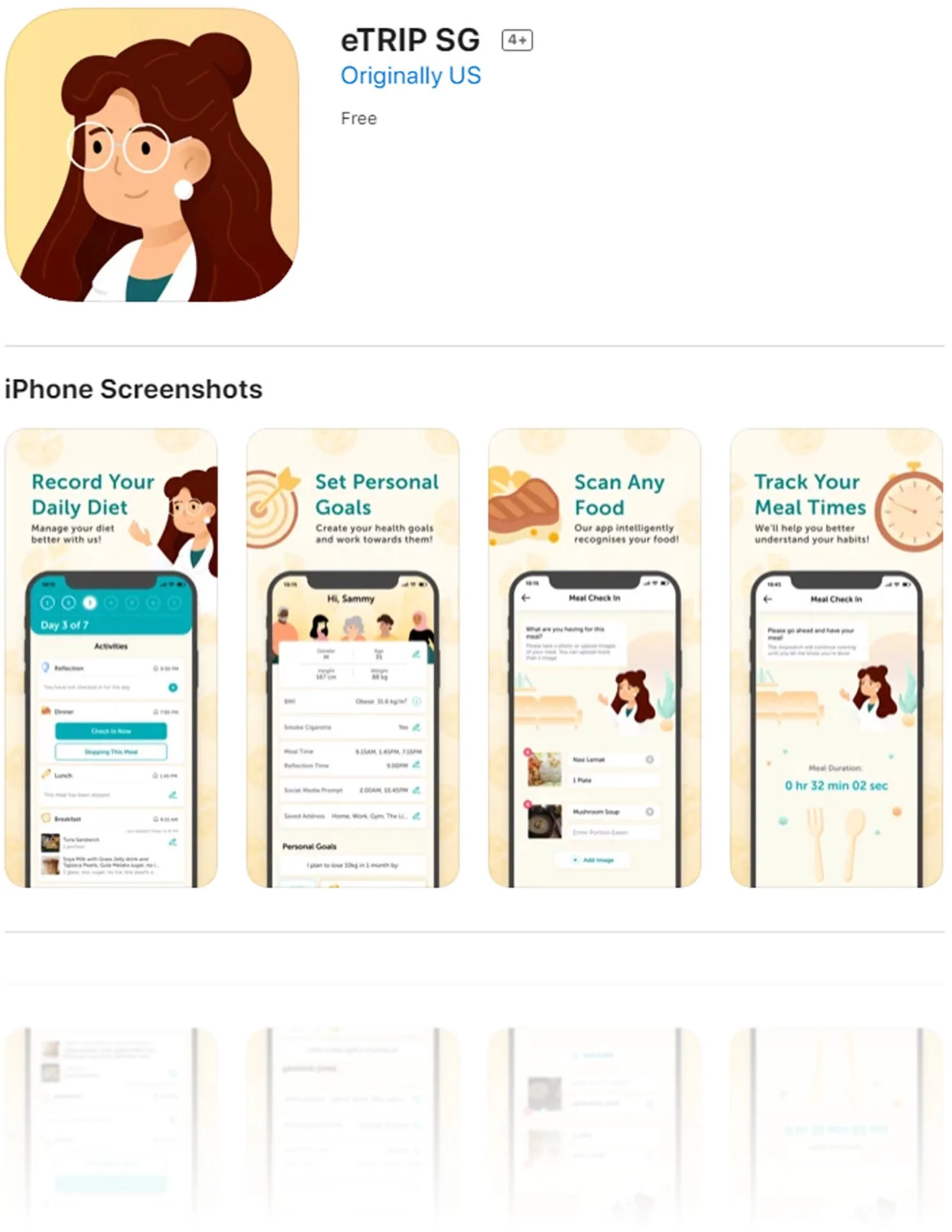
Screen shot of the eTRIP app.

We developed a mobile behavior change application (eTRIP 2.0) designed to support weight loss among young adults with excess body weight by strengthening core self-regulatory skills. The intervention integrates a self-regulation training framework with image-based food logging (enhanced with computer recognition technology), dietary check-in nudging, psychoeducational content, and social support (Figure 2).

eTRIP 2.0 builds upon an earlier version of the app (eTRIP 1.0), which consisted primarily of image-based food logging and dietary check-in nudging and had previously been shown to improve eating habits, physical activity, and mental well-being.^26^ The upgraded version was intentionally designed to move beyond self-monitoring alone by incorporating structured self-regulation training components aimed at addressing the intention-behaviour gap.

### Self-regulation framework and strategies

The self-regulation framework underpinning eTRIP 2.0 was developed based on temporal self-regulation theory, which posits that health behaviors are shaped by the dynamic interplay between intentions, self-regulatory capacity, and prepotent contextual cues, particularly in environments characterized by immediate rewards and competing demands. Within this framework, sustained behavior change depends not only on motivation but also on the ability to translate intentions into action through effective self-regulatory processes over time. Accordingly, eTRIP 2.0 operationalizes self-regulation as a cyclical process involving goal awareness, self-monitoring, feedback, reflection, emotional regulation, and adaptive action planning. Specifically, the intervention targets the following self-regulatory strategies: (1) self-monitoring of eating behavior and lapse triggers; (2) reflective self-evaluation of progress, thoughts, and emotions; (3) emotional regulation through acceptance-based strategies; (4) implementation intentions using if-then planning; and (5) adaptive planning to refine strategies based on prior experiences. These strategies and their delivery within the app are detailed in Table 1.

**Table 1:** Mapping of temporal self-regulation theory constructs, self-regulation strategies, intervention components, and behavior change techniques taxonomy (BCTT version 1) in eTRIP 2.0.

TST construct	Self-regulation strategy	Description	Implementation in eTRIP 2.0	BCTT code(s)	BCT label(s)
**Self-regulatory capacity**	Self-monitoring	Increasing awareness of eating behavior and lapse triggers	Image-based food logging and dietary check-in using eBLISS at each meal	2.3	Self-monitoring of behavior
**Self-regulatory capacity**	Feedback	Comparing current behavior against goals	Automated visual feedback on logged meals and weekly summaries	2.2	Feedback on behavior
**Intention**	Reflective self-evaluation	Reviewing progress, thoughts, emotions, and challenges	Guided weekly reflection on dietary problems, goals, thoughts, and feelings	2.4	Self-monitoring of outcomes
**Prepotent cues**	Emotional regulation	Managing affect-driven eating impulses	Animated psychoeducation on observing and accepting negative emotions without compensatory eating	11.2	Reduce negative emotions
**Intention × context**	Problem solving	Identifying barriers and facilitators of goal attainment	Weekly reflection prompts on lapse triggers and contextual challenges	1.2	Problem solving
**Intention → behavior**	Implementation intentions	Automating goal-consistent responses in high-risk situations	If-then action planning for anticipated lapses	1.4	Action planning
**Self-regulatory capacity**	Adaptive planning	Updating strategies based on prior experience	Selection of self-regulation strategies for the following week	1.5	Review behavior goals
**Prepotent cues**	Prompts and cues	Maintaining goal salience at moments of vulnerability	Meal-time push notifications for food logging	7.1	Prompts/cues
**Social influences**	Social support	Reinforcing self-regulation through peer interaction	In-app social support features	3.1	Social support (unspecified)
**Knowledge support**	Instruction	Supporting informed dietary decisions	Infographics on portion size and balanced meals (“My Healthy Plate”)	4.1	Instruction on how to perform the behavior

### Dietary education and scope of weight loss training

Participants were not prescribed daily calorie targets nor explicitly trained to calculate energy deficits. Instead, the intervention prioritized the development of self-regulatory capacity rather than prescriptive calorie restriction. Foundational dietary education was provided through infographics and animated images based on the national “My Healthy Plate” guideline, which emphasizes portion size estimation and balanced meal composition (a 10-inch plate with one-quarter whole grains, one-quarter proteins, and half fruits and vegetables).^27^ This approach was intended to support informed decision-making without imposing rigid dietary rules.

### App features and intervention delivery

At participants’ pre-set mealtimes, automated prompts were delivered to cue food logging. Participants were nudged to photograph their food items and complete a brief dietary check-in using the Eating Behavior Lapse Inventory Survey Singapore (eBLISS), which assesses common dietary lapse triggers such as emotions and eating context.^28^ This process typically required 1-2 minutes per meal. Food images were analyzed using a locally developed artificial intelligence-assisted food image recognition system, which automatically generated estimated calorie counts that were logged in the backend to support awareness, reflection, and goal enforcement.

At the end of each week, participants were prompted to review their food logs and complete a guided weekly reflection focusing on dietary challenges, goals, thoughts, emotions, and perceived progress. Based on this reflection, participants selected from a set of previously developed self-regulation strategies and formulated personalized implementation intentions using an if-then logic model to guide behavior in anticipated high-risk situations for the subsequent week.^29^ These strategies aimed to enhance psychological flexibility and adaptive self-regulation in response to contextual and emotional eating triggers.

Psychoeducational content on emotional regulation was delivered using animated visuals illustrating the process of observing and accepting negative emotions as transient experiences that need not be acted upon through compensatory behaviors such as eating. Social support was facilitated through in-app features that allowed participants to engage with peers undergoing the intervention.

### Intervention components

Intervention delivery was fully computer-programmed, ensuring consistency and fidelity across participants. Intervention components were mapped to the Behaviour Change Technique Taxonomy v1 (BCTTv1),^30^ including self-monitoring, action planning, problem solving, prompts/cues, and emotional regulation techniques; this mapping is presented in Table 1.

### Outcomes

The primary outcome was weight, while the secondary outcomes were self-regulation, nutrition knowledge, eating phenotypes, overeating habits, anxiety, and depression.

Secondary outcomes were selected a priori based on temporal self-regulation theory, which conceptualizes health behavior as the interaction between intentions, self-regulatory capacity, and prepotent cues, as well as the extent to which behaviors become automatic over time. Self-regulation of eating behavior (SREBQ) was included to assess changes in self-regulatory capacity targeted by the intervention. Eating behavior patterns (uncontrolled eating, cognitive restraint, and emotional eating) and overeating automaticity (Self-Report Behavioral Automaticity Index [SRBAI]) were measured to capture the influence of affective and contextual prepotent cues and habitual processes on eating behavior. Nutrition knowledge was assessed as an upstream determinant supporting intention formation. Anxiety and depressive symptoms were included, given their role in depleting self-regulatory capacity and amplifying cue-driven eating. App engagement reflected exposure to self-regulatory support. Body composition measures complemented weight outcomes by capturing downstream physiological correlates of sustained behavior change.

*Weight (primary outcome)*, BMI, body fat mass, body fat percentage, basal metabolic rate, waist-hip ratio, and visceral fat level were assessed using the InBody 120 (Seoul, Korea) body composition analyzer (BCA), which has been used in various studies for BCA measurements.^31^^,^^32^ Height was assessed only at baseline using a measuring tape stuck to the wall. The research assistant ensured that the BCA was calibrated to zero at every weigh-in to ensure outcome data quality.

*Self-regulation of eating behavior* was assessed using the Self-Regulation of Eating Behavior Questionnaire (SREBQ). It comprises 5 main items measured on a 5-point Likert scale. The total mean score was calculated, and higher scores represent higher levels of self-regulation. Mean scores categorize self-regulation into low (<2.8), medium (2.8-3.6), and high (>3.6).^33^ An example item is, “I’m good at resisting tempting food.” The SREBQ also includes 4 screening questions on healthy diet intentions (eg, “Do you intend to have a healthy diet?”). With reference to the UK adult population, reliability of the SREBQ is high, with an *α* of 0.75 and test-retest reliability at 0.77. Construct validity is well-supported by positive correlations with general self-regulation measures and negative correlations with food responsiveness and emotional overeating (*P* < .001).^33^

*Nutrition knowledge* was assessed using the General Nutrition Knowledge Questionnaire-Revised (GNKQ-R), which comprises 88 questions scored against an answer sheet. The questions can be categorized into 5 subscales—Dietary recommendations, Food groups, Food choices, Food labels and Diet, disease and weight association. Each correct answer received a score of 1 point, adding up to a total maximum score of 88.^34^ Higher scores represent a higher level of nutrition knowledge. The GNKQ-R demonstrated high internal reliability (Cronbach’s *α* = .93) and test-retest reliability, with intraclass correlation coefficients ranging from 0.72 to 0.89 across subscales, indicating strong consistency and stability. Validity was confirmed through known-groups and convergent validity methods, with significantly higher scores among dietetics students than English students (effect size *d* = 1.2, *P* < .001).^34^ The tool was contextualized by changing the example of a healthy diet guideline in section 1, question 9 from “Eatwell guide” to “My Healthy Plate” and “starchy foods” to “whole grains.” The right answer for section 4 question 14 on the BMI classification of 23 kg/m^2^ was also contextualized from “normal” to “overweight” to suit the Asian cut-off scores.^35^

*Eating Behavior* was assessed using the Three-Factor Eating Questionnaire (TFEQ), which has 3 subscales—Uncontrolled Eating Scale with 9 items, Cognitive Restraint Scale with 6 items, and Emotional Eating Scale with 3 items.^36^ An example of a TFEQ item is, “when I feel nervous, I find myself eating.” Raw scale scores are calculated for each subscale by taking the mean of all items in each scale multiplied by the number of items in the scale. Raw scores are then transformed to a zero to 100 scale, where higher values indicate more uncontrolled eating, cognitive restraint or emotional eating. Initially developed for an obese population, the TFEQ has demonstrated high internal consistency (*α* = 0.85-0.93) and strong test-retest reliability (*α* = 0.80-0.93). Validity has been confirmed through correlations with binge eating and overeating in response to emotional and social cues.^36^

*Automaticity or habit of overeating behavior* was assessed using the SRBAI, which comprises 4 items measured on a 7-point Likert scale. The mean scores across all items were calculated and taken as the final score, where higher scores suggest a stronger habit of overeating.^37^ An example of an SRBAI item is, “Behavior ___ is something I do automatically.” In a more recent validation study, the SRBAI shows high reliability (*α* = 0.93) and excellent validity, with each item being strongly correlated (*r* = .85-.94, *P* < .05) with the overall scale and behavior frequency, confirming its effectiveness in measuring automaticity in habits.^38^

*Feasibility outcomes* were assessed as tertiary outcomes and included participant retention at weeks 12 and 24, as well as intervention engagement, measured by the proportion of scheduled dietary check-ins completed during the intervention period (at least 3 times a day). Participants’ check-in rates were dichotomized into high and low by average split.

*Anxiety* was assessed using the Generalized Anxiety Disorder 2-item (GAD-2). Each item has a scale range of 0-3, with total scores ranging from 0 to 6. Higher scores represent higher levels of anxiety.^39^ An example of a GAD-2 item is, “Over the last 2 weeks, how often have you been bothered by the following problems? (Feeling nervous, anxious or on edge).” With reference to adults in Australia, the GAD-2 has demonstrated good reliability (*α* = 0.81) and test-retest reliability of 0.81.^40^ Validity is supported by its correlation with the GAD-7 and its ability to discriminate between individuals with and without an anxiety diagnosis, showing acceptable sensitivity and specificity at a cut-off score of ≥3 (sensitivity = 0.71, specificity = 0.69).^40^

*Depression* was assessed using the Patient Health Questionnaire 2-item (PHQ-2). Each item has a scale range of 0-3, with total scores ranging from 0-6. Higher scores represent higher levels of depression.^41^ An example of a PHQ-2 item is, “Over the last 2 weeks, how often have you been bothered by any of the following problems? (Feeling down, depressed, or hopeless)”. When evaluated on medical outpatients in Germany, the PHQ-2 has demonstrated good internal consistency (*α* = 0.83). Its criterion validity is supported by a sensitivity of 87% and a specificity of 78% for detecting major depressive disorder at a cut-off score of ≥3, in comparison with the Structured Clinical Interview for DSM-IV.^42^

### Data analysis

Data analysis was conducted using IBM SPSS 29^43^ and R.^44^ Data entry was verified by 2 independent study team members to ensure data quality. Sociodemographic characteristics were summarized using mean (SD), median (IQR), and count (%). Baseline group differences were assessed using the chi-square test of independence for categorical variables and the independent sample *t*-test for continuous variables. The intention-to-treat (ITT) approach was used with missing values imputed using mean or median when appropriate. Adjusted group time interactions on primary and secondary outcomes were assessed using generalized estimating equations (GEE) to account for repeated-measures correlations. In contrast to an independent *t*-test, which compares mean differences between 2 independent groups at a single time point, the GEE allows for the simultaneous estimation of group, time, and group-by-time interaction effects while adjusting for potential covariates. It also accommodates data missing at random and supports various outcome distributions (eg, continuous, binary, count) through appropriate link functions, coherent with the ITT approach. Thus, the use of GEE provides a more flexible and statistically rigorous method for evaluating longitudinal or clustered data in this study. Multiple linear regression was used to examine the association between the secondary outcomes and changes in weight and self-regulation.

## Results

### Baseline characteristics

A total of 302 participants were assessed for eligibility, of which 146 of them were excluded with reasons (Figure 1). A total of 156 participants were recruited from November 14, 2023, to October 27, 2024 (Figure 1). Sociodemographic charactiersitcs of the participants are detailed in Table 2. At baseline, the mean age, weight, BMI, waist circumference, and Body Roundness Index (BRI) of the participants were 25.9 ± 4.1 years old, 75.9 (IQR = 17.6) kg, 26.1 (IQR = 4.5) kg/m^2^, 86.8 ± 11.0 cm, and 3.5 ± 1.2. The sociodemographic characteristics, baseline anthropometric, and psychological construct measures are shown in Tables 1 and 2, respectively. Only 15.4% and 46.2% of the participants had experience with using weight loss apps and smartwatches, respectively. There was a slightly higher representation of Chinese (85.3%) in our sample as compared to our national demographic of 75.6%.^45^ There were no significant differences between the intervention and the control groups (Tables 2 and 3), and the attrition rate for the study was 6.4% and 12.2% at weeks 12 and 24, respectively.

**Table 2:** Sociodemographic characteristics, experience with using apps and smartwatches of the 156 participants who completed the study and group differences between the intervention and control groups.

Characteristics	Mean ± SD/frequency (%)			*P*-value
	All (*n* = 156)	Intervention group (*n* = 78)	Control group (*n* = 78)	
**Age (years), mean ± SD**	25.9 ± 4.1	26.2 ± 4.3	25.5 ± 3.9	.29
**Sex, *n* (%)**				.63
** Males**	87 (55.8)	45 (57.7)	42 (53.8)	
** Females**	69 (44.2)	33 (42.3)	36 (46.2)	
**Race, *n* (%)**				.90
** Chinese**	133 (85.3)	67 (85.9)	66 (84.6)	
** Indian**	11 (7.1)	6 (7.7)	5 (6.4)	
** Malay**	8 (5.1)	3 (3.8)	5 (6.4)	
** Others**	4 (2.6)	2 (2.6)	2 (2.6)	
**Marital status, *n* (%)**				.65
** Single**	151 (96.8)	75 (96.2)	76 (97.4)	
** Married**	5 (3.2)	3 (3.8)	2 (2.6)	
**Religion, *n* (%)**				.96
** Buddhism**	39 (25.0)	19 (24.4)	20 (25.6)	
** Christianity**	44 (28.2)	21 (26.9)	23 (29.5)	
** Hinduism**	6 (3.8)	3 (3.8)	3 (3.8)	
** Islam**	9 (5.8)	4 (5.1)	5 (6.4)	
** Free thinker**	55 (35.3)	30 (38.5)	25 (32.1)	
** Others**	3 (1.9)	1 (1.3)	2 (2.6)	
**Highest education level, *n* (%)**				.49
** Pre-university**	48 (30.8)	26 (33.3)	22 (28.2)	
** University**	108 (69.2)	52 (66.7)	56 (71.8)	
**Per-capita household income (S$/month), *n* (%)**				.83
** <1000**	14 (8.5)	8 (10.3)	6 (7.7)	
** 1000-3000**	34 (21.8)	15 (19.2)	19 (24.4)	
** 3001-5000**	39 (25.0)	20 (25.6)	19 (24.4)	
** 5001-10000**	44 (28.2)	24 (30.8)	20 (25.6)	
** >10 000**	25 (16.0)	11 (14.1)	14 (17.9)	
**Employment, *n* (%)**				.44
** Full-time**	92 (59.0)	46 (59.0)	46 (59.0)	
** Part-time**	12 (7.7)	4 (5.1)	8 (10.3)	
** Unemployed**	52 (33.3)	28 (35.9)	24 (30.8)	
**Experienced using weight loss apps, *n* (%)**				.66
** Yes**	24 (15.4)	13 (16.7)	11 (14.1)	
** No**	132 (84.6)	65 (83.3)	67 (85.9)	
**Experienced using smartwatches, *n* (%)**				.75
** Yes**	72 (46.2)	37 (47.4)	35 (44.9)	
** No**	84 (53.8)	65 (83.3)	78 (55.1)	

**Table 3:** Baseline anthropometric and psychological construct measures of the 156 participant who completed the study and group differences between the intervention and control groups.

Characteristics	Mean ± SD/frequency (%)			Test-statistic, *P*-value
	All (*N* = 156)	Intervention group (*n* = 78)	Control group (*n* = 78)	
**Anthropometric measures**				
**Weight (kg), median, IQR**	75.9, 17.6	76.5, 20.4	73.5, 13.1	.20
**BMI (kg/m^2^), median, IQR**	26.1, 4.5	26.3, 4.3	25.6, 4.5	.66
**BMI classifications,^a^ *n* (%)**				
**Overweight (23.0-27.4 kg/m^2^; moderate risk)**	98 (62.4)	49 (62.8)	49 (62.8)	-
**Obese I (27.5-32.4 kg/m^2^; high risk)**	45 (28.7)	21 (26.9)	24 (30.8)	-
**Obese II (32.5-37.4 kg/m^2^; very high risk)**	9 (5.7)	6 (7.7)	30.8 (3.8)	-
**Obese III (≥37.4 kg/m^2^; very high risk)**	4 (2.5)	2 (2.6)	2 (2.6)	-
**Waist circumference (cm), median, IQR**	86.8-11.0	87.1-13	86-9.6	.29
**Body fat mass (kg), median, IQR**	21.8-9.3	22.0-9.5	21.2-9.3	.99
**Body fat % (%), median, IQR**	29.5-13.5	28.9-13.2	29.9-14.3	.53
**Skeletal muscle mass (kg), median, IQR**	30.8-10.9	31.3-12.5	30.5-9.3	.12
**Basal metabolic rate (kcal), median, IQR**	1548.5-393	1552.5-450	1545.5-325	.13
**Visceral fat level, median, IQR**	9-5	9-5	9-5	1.00
**BRI, median, IQR**	3.5-1.2	3.4-1.1	3.7-1.2	.91
**Nutrition knowledge (GNKQ-R)**	61.5 ± 9.1	61.6 ± 9.8	61.45 ± 8.28	.85
**Psychological flexibility (AAQW)**	80.5 ± 21.0	81.15 ± 22.2	79.14 ± 20.1	.55
**Eating phenotypes (TFE)**				.51
**Uncontrolled eating**	47.4 ± 22.1	48.5 ± 22.0	46.2 ± 22.27	.53
**Cognitive restraint**	45.0 ± 19.5	44.1 ± 19.3	46.01 ± 19.31	.66
**Emotional eating**	45.8 ± 30.2	47.2 ± 30.7	45.01 ± 30.28	.17
**Eating self-regulation (SREBQ)**	2.96 ± 0.57	2.9 ± 0.59	3.04 ± 0.55	.76
**Overeating habit (SRBAI)**	3.63 ± 1.70	3.8 ± 1.68	3.54 ± 1.71	.23
**Future thinking (CFC-6)**	4.79 ± 1.11	4.7 ± 1.23	4.89 ± 0.96	.45
**Anxiety (GAD-2)**	2.26 ± 1.84	2.3 ± 1.9	2.19 ± 1.81	.38
**Depression (PHQ-2)**	1.61 ± 1.70	1.7 ± 1.7	1.38 ± 1.7	.09

a BMI classification was derived from the recommended Asian cut-off scores.^65^

Abbreviations: AAQW, The Acceptance and Active Questionnaire for Weight-Related Difficulties; CFC-6 consideration of future consequences 6-items; GAD-2, Generalized Anxiety Disorder 2-item; GNKQ-R, General Nutrition Knowledge Question-revised; PHQ-2, Patient Health Questionnaire 2-item; SREBQ, Self-regulation of Eating Behavior Questionnaire; SRBAI, Self-Report Behavioral Automaticity Index; TFEQ, The Three-Factor Eating Questionnaire.

### Group difference in weight and self-regulation

**Figure 3:**
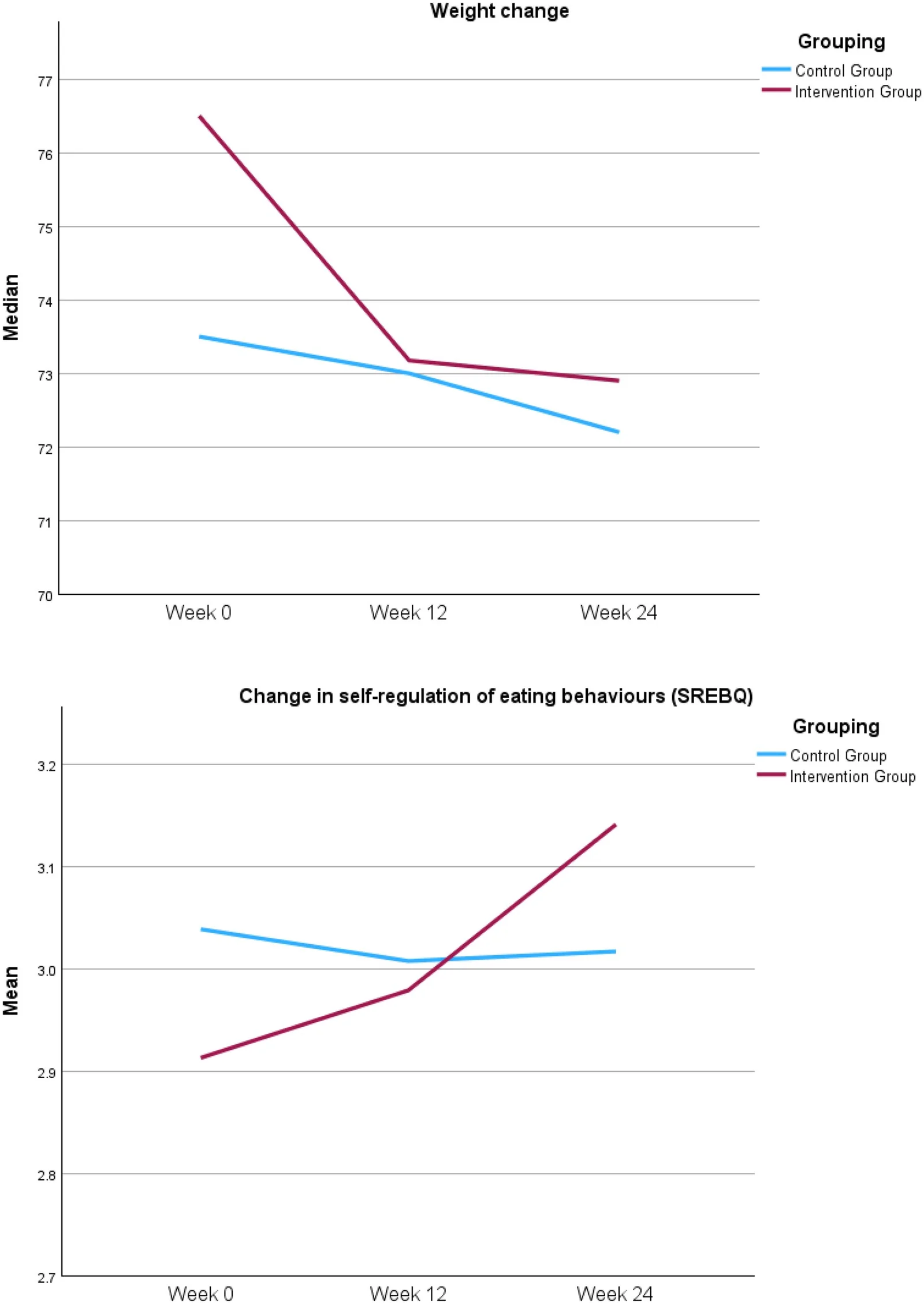
Illustrations of the mean changes in weight and self-regulation of eating behavior over weeks 0, 12, and 24.

We observed a consistent decrease in weight over 6 months and an increase in self-regulation among the participants in the intervention group, while there were fluctuations in weight among those in the control group (Table 4). Significant group differences were found for weight change at week 12 and self-regulation at week 24 (Table 5, Figure 3).

**Table 4:** Group differences in weight and self-regulation.

	Intervention group			Control group		
Variables	Baseline	Week 12	Week 24	Baseline	Week 12	Week 24
**Weight, kg**						
**Mean, mean (SD)**	78.3 kg (13.8)	76.4 (12.4)	74.7 (14.7)	75.7 (11.8)	76.5 (11.1)	75.9 (10.5)
**Median, median (range)**	76.5 (59.1)	76.3 (59.0)	75.3 (61.7)	73.5 (59.1)	76.3 (59)	75.3 (61.7)
**Self-regulation**						
**Mean, mean (SD)**	2.9 (0.59)	2.98 (0.53)	3.14 (3.08)	3.0 (0.54)	3.0 (0.56)	3.0 (0.52)

**Table 5:** Group changes in weight and self-regulation at week 12 (T2) and week 24 (T3).

	Unadjusted model		Adjusted model^a^	
	B (95% CI)	*P*-value	B (95% CI)	*P*-value
**Weight (kg)**				
**Intervention group**	2.6 (2.04 to −1.38)	.20	2.70 (−1.40 to 6.77)	.20
**T3**	−0.388 (−0.25 to 0.69)	.25	−0.38 (−1.03 to 0.27)	.25
**T2**	0.22 (−0.25 to 0.69)	.36	0.22 (−0.25 to 0.69)	.36
Intervention group * T3	−2.0 (−4.98 to 0.99)	.19	−2.01 (−5.00 to 0.97)	.19
Intervention group * T2	−0.80 (−1.51 to −0.09)	.027	−0.81 (−1.50 to −0.10)	.025
**Self-regulation**				
**Intervention group**	−0.13 (−0.30 to 0.05)	.17	−0.14 (−0.32 to 0.04)	.14
**T3**	−0.05 (−0.15 to 0.06)	.42	−0.05 (−0.16 to 0.06)	.41
**T2**	−0.05 (−0.14 to 0.05)	.34	−0.05 (−1.14 to 0.05)	.33
Intervention group * T3	0.25 (0.08 to 0.42)	.004	0.25 (−0.01 to 0.02)	.52
Intervention group * T2	0.09 (−0.07 to 0.24)	.29	0.09 (−0.07 to 0.24)	.29

a Adjusted for age, and sex.

### Association between secondary outcomes

At baseline, weight was not significantly correlated with any of the secondary outcomes measured including eating phenotype, time perspective, overeating habits, anxiety and depressive symptoms. Self-regulation was significantly correlated with all the secondary outcomes. We tested 3 models to predict each of the 3 eating phenotypes. Time perspective, overeating habit, self-regulation, and anxiety and depressive symptoms explained 77.6% of uncontrolled eating, 40.5% of cognitive restraint, and 68.5% of emotional eating (Table 6).

**Table 6:** Multiple regression analysis of eating phenotypes on time perspective, overeating habit, anxiety symptoms, depression symptoms and self-regulation.

Variables	Unstandardized B	Standard error	*t*	*P*-value
**Uncontrolled eating**				
**Time perspective**	−1.33	1.27	−.06	.30
**Overeating habit**	6.69	.81	.51	<.001^a^
**Anxiety**	1.96	.81	.16	.02^b^
**Depression**	.75	.89	.06	.40
**Self-regulation**	−7.84	2.48	−.20	.002^c^
**Cognitive restraint**				
**Time perspective**	1.08	1.60	.69	.50
**Overeating habit**	−.49	1.02	.68	.63
**Anxiety**	1.2	1.02	−.48	.24
**Depression**	−1.40	1.12	1.17	.21
**Self-regulation**	11.27	3.12	−1.26	<.001^a^
**Emotional eating**				
**Time perspective**	−1.420	2.014	−.705	.482
**Overeating habit**	6.025	1.285	4.687	<.001^a^
**Anxiety**	4.572	1.288	3.551	<.001^a^
**Depression**	−1.082	1.406	−.770	.443
**Self-regulation**	−14.880	3.929	−3.787	<.001^a^

a *P*-value ≤.001,

b ≤.05,

c ≤.01.

### Difference in adherence and impact on weight

Among participants in the intervention group, those with high check-in adherence (*n* = 44, >60%) lost 2.73 kg compared to −0.97 kg among those with low adherence (*n* = 34) but this difference was not statistically significant.

## Discussion

This study partially supported the prespecified hypotheses, with effects differing by outcome and time point. At the primary end-of-intervention time point (week 12), participants receiving the digital self-regulation intervention demonstrated significantly greater weight loss compared with controls, supporting the hypothesis that the intervention would improve weight-related outcomes during active exposure. However, group differences in weight were attenuated by week 24 following removal of access to the intervention, indicating that the weight loss effect was not fully sustained over time. In contrast, improvements in eating self-regulation did not differ significantly between groups at week 12 but emerged at week 24, supporting the hypothesis of improved self-regulation at follow-up rather than immediately post-intervention. This temporal pattern suggests that behavioral and anthropometric outcomes responded on different timelines, ­consistent with TST, whereby behavioral change may precede conscious improvements in self-regulatory capacity.

### Improvements in weight loss

Amidst the mixed findings of existing literature on the effectiveness of app-based weight management programs,^46^ we demonstrated a 2 kg higher weight loss among participants who had used our digital self-regulation intervention, potentially due to the focus on self-regulation. This is in comparison to prior research that showed only a 0.87 kg weight loss difference at the end of using a 12-week psychoeducational app^47^ and another using MyFitnessPal for 6 months, which showed no significant weight difference between the 2 groups.^48^ Consistent with a systematic review, group differences in weight loss from week 12 to 24 attenuated, potentially due to the common phenomenon that weight loss tends to peak at 3 months, tapering off with time and the removal of digital health interventions.^49^ This was expected and points toward the need for long-term, resource-efficient interventions like apps for new behaviors to be sustained automatically as habits or to have a human health coach to provide accountability to sustain motivation and engagement with the program (as mentioned as feedback by some participants).^50^ While this is commonly seen as a limitation of weight loss apps, the non-inferiority effect on weight loss compared to multidisciplinary face-to-face obesity counseling sessions could be a more cost-effective way to manage obesity, especially in those with less severe obesity and when strategically targeted at people with high motivation and engagement.^51^

### Clinical significance of weight loss

Beyond statistical significance, the clinical relevance of the observed weight change warrants consideration. In behavioral weight management, modest weight reductions of approximately 3%-5% of baseline body weight have been consistently associated with clinically meaningful improvements in cardiometabolic risk factors, including glycaemic control, blood pressure, and lipid profiles.^52^ In the present study, the magnitude of weight loss observed in the intervention group meets or approaches these commonly accepted benchmarks, suggesting potential clinical benefit despite the modest absolute reduction. This finding is ­particularly relevant in young adults, for whom early weight reduction may confer disproportionate long-term benefits by reducing cumulative exposure to excess adiposity and associated metabolic risk across the life course. Taken together, these results indicate that the observed weight loss is not only statistically ­significant but also clinically meaningful within the context of early, scalable obesity interventions.

### Feasibility of the digital self-regulation intervention

While the average app engagement rate of the intervention group participants was 60%, our attrition rate was 12.2%, which is a relatively low number for a behavior change intervention that averages out at 40%.^53^ The higher weight loss among individuals with higher app engagement also coincides with existing studies on the positive relationship between app engagement and weight loss, though non-significant in this instance.^54^ While causal inference cannot be made, several factors may plausibly have contributed to the low attrition rate. From an intervention perspective, eTRIP 2.0 was designed to minimize participant burden through brief, meal-time interactions (typically 1-2 minutes), automated prompts, and fully app-based delivery, which may have facilitated sustained engagement. In addition, the intervention emphasized self-regulation and psychological flexibility rather than prescriptive calorie targets, which may have reduced perceived burden and disengagement. From a population perspective, the study targeted young adults who are generally comfortable with smartphone-based interventions and image-based interactions, which may have further supported retention. These feasibility metrics suggest that the intervention was acceptable and manageable for participants.

### Understanding how digital self-regulation interventions improve weight loss

Surprisingly, weight and weight change were not significantly associated with the secondary outcomes measured. This finding echoes our earlier observation that overeating habits were not directly linked to body weight, underscoring the complex and nonlinear relationship between behavioral, psychological, and physiological change.^55^ Weight is a delayed and imperfect proxy of eating behavioral modification, influenced by homeostatic and metabolic compensations that can mask short-term improvements. Even when individuals begin to modify their eating patterns, underlying physiological adaptations such as adaptive thermogenesis and metabolic efficiency may limit the detectability of weight loss. Moreover, fluctuations in hydration, glycogen, and hormonal balance introduce day-to-day variability, making weight a noisy indicator of progress in short-duration interventions.

### Temporal lag in self-regulation improvement

Interestingly, in trying to uncover the mechanism behind the weight change, we found a delay in the response to a change in self-regulation scores, as the difference in scores between the 2 groups only appeared at week 24, when the difference in weight loss became statistically non-significant. We speculate that this could have been due to the longer time needed to internalize and be self-aware of the change in self-regulation, a lack of sensitivity of the SREBQ, or a lack of power in the sample size to detect a change.^56^^,^^57^

From a behavioral science standpoint, this lack of association also reflects the asynchronous timing of change processes, where improvements in self-regulation capacity typically emerge later in the change trajectory. Early in an intervention, individuals may display motivational engagement and modest behavioral and anthropometric improvements (eg, reduced frequency of overeating) driven by heightened awareness or external cues, without yet achieving the sustained self-regulatory strength necessary for long-term maintenance. Self-regulation is akin to a muscle that strengthens gradually through repeated effortful practice; it often improves after initial behavioral changes have been attempted, relapsed, and reattempted.^6^ Future studies could examine these processes using lagged analyses or latent growth modeling to better elucidate how self-regulation capacity evolves and mediates long-term weight outcomes.

### Weight loss in both groups potentially due to the question-behavior effect

Weight reduction observed in both the intervention and control groups may, in part, be attributed to the question-behavior effect (QBE), also known as the mere measurement effect. This phenomenon occurs when the act of measuring anthropometry and asking participants about their behaviors, attitudes, or intentions induces self-reflection and increases awareness, leading to short-term behavior change even in the absence of an active intervention.^58^^,^^59^ Prior studies have shown that completing health-related surveys can prompt individuals to initiate healthier behaviors.^60^ Thus, the baseline assessment itself may have acted as a low-intensity behavioral cue, partially explaining the modest but parallel weight changes across study arms.

Frequent self-weighing allows participants to observe how their behavior affects their weight and enables them to adjust their behavior to prevent weight gain.^58^ While this can be considered a confounding factor in the current study, it also highlights the importance of regular body composition analysis as a facilitator of weight loss, as many participants indicated that they were particularly looking forward to the next outcomes measurement to monitor their progress in weight and fat loss. Weight management programs especially clinical ones could hence consider including BCAs into the program package to encourage self-monitoring, motivation, and weight loss maintenance. Future long-term studies could explore the effectiveness of simply conducting mass measurement of body composition to promote obesity management.

### Strengths and limitations

This study has several notable strengths that add insights to the limited literature on standalone mobile apps that focus on enhancing behavioral change through self-regulation. Firstly, while previous studies have evaluated the effectiveness of mobile app interventions, this is the first study to examine the use of a TST-guided app designed to support weight loss among young adults with excess body weight. Secondly, this study showed the potential for scalability in a community setting, as the app-based weight loss support requires less manpower than traditional clinical weight management programs, which makes it more accessible and sustainable for the healthcare system.^61^ Thirdly, this study evaluates behavioral and psychological outcomes aside from anthropometric outcomes, providing a comprehensive assessment of the app’s impact on various aspects of health and well-being that contribute to weight loss.

However, this study has several limitations. Firstly, the use of convenience sampling could have resulted in selection bias and limited generalizability beyond the sample.^62^ Future trials could consider employing probability sampling for broader applicability. Secondly, due to the blinding of the participants, some participants in the control group thought that the body composition measurement was an intervention. This potentially resulted in subjective bias in patients’ participation and reporting of the outcome, especially on self-report subjective measures such as nutrition knowledge, psychological flexibility, and eating behaviors.^63^ Thirdly, participants were not required to fast before the weigh-ins, which could have potentially confounded our findings. Lastly, as the sample size was not powered based on the secondary outcomes, it may not have been powered enough to detect statistically significant changes.

## Conclusion

In conclusion, our study showed that even a standalone self-regulation app-based program provides significant benefits on anthropometric outcomes among people with excess adiposity, demonstrating its strong potential as a scalable, sustainable, resource-efficient adjuvant behavior change intervention to existing weight management programs. Such digital self-regulation programs could be used to augment and reduce the resource intensity of health coaching, which typically requires frequent client engagements to achieve a modest amount of weight loss. Using a phone app could also enhance access to weight management programs, especially among community-dwelling individuals living with obesity who are not ready for a clinic consultation.^64^ Future studies could consider testing the implementation of such interventions in the community to ascertain their implementation outcomes.

## Data Availability

The datasets generated or analyzed during this study are available from the corresponding author on reasonable request.
